# A Pilot Study on the Association between Cardiovascular Risk Factors and Coronary Artery Calcification in a Group of Patients Investigated via Cardiac Computed Tomography in a European Country with High Cardiovascular Risk

**DOI:** 10.3390/biomedicines11112926

**Published:** 2023-10-30

**Authors:** Adriana Sorina Capisizu, Silviu Marcel Stanciu, Dragos Cuzino

**Affiliations:** 1 Faculty of General Medicine, Carol Davila University of Medicine and Pharmacy, 8 Eroii Sanitari Bvd., 050474 Bucharest, Romania; 2 Center for Cardiovascular Diseases, Laboratory of Noninvasive Cardiovascular Functional Explorations, Central Military Emergency University Hospital “Dr. Carol Davila”, 134 Calea Plevnei Str., 010825 Bucharest, Romania; 3 Clinical Radiology-Medical Imaging Center, Central Military Emergency University Hospital “Dr. Carol Davila”, 134 Calea Plevnei Str., 010825 Bucharest, Romania

**Keywords:** cardiac computed tomography, risk factors, calcium score, cardiovascular disease, coronary artery disease

## Abstract

(1) Background: Cardiovascular disease is the leading cause of mortality worldwide; the prevention and early detection of coronary artery disease are of critical importance; and the coronary artery calcium score is a powerful method in the assessment of coronary artery disease. Among European countries, Romania ranks as a country with a very high risk of cardiovascular diseases, but the data are limited in regard to the prevalence of the calcium score. (2) Methods: A retrospective study was conducted to establish the coronary calcium score in a group of patients investigated via cardiac CT and to determine the correlation with the presence of cardiovascular risk factors. (3) Results: According to the Agatston calcium score, 50% of the patients had a positive calcium score. High calcium scores above 400 UA were present in 12.6% of patients. Regarding the association between the presence of cardiovascular risk factors and the levels of coronary artery calcification, a mild level of calcification was associated with age over 50 years (X^2^ = 3.88, *p* = 0.04, OR = 3.25; 95% CI 0.94–11.14); a moderate level of calcification with the age of patients over 50 years (X^2^ = 6.54, *p* = 0.01, OR = 5.58; 95% CI 1.29–24.16), dyslipidemia (X^2^ = 7.28, *p* = 0.007, OR = 3.37; 95% CI 1.34–8.51), and arterial hypertension (X^2^ = 5.37, *p* = 0.02, OR = 2.88; 95% CI 1.14–7.27); a severe level of calcification with hypertension (X^2^ = 4.61, *p* = 0.03, OR = 7.03; 95% CI 0.90–54.81); and a very severe level of calcification with hypertension (X^2^ = 4.61, *p* = 0.03, OR = 7.03; 95% CI 0.90–54.81), smoking (X^2^ = 8.07, *p* = 0.004, OR = 4.44; 95% CI 1.47–13.44), and diabetes (X^2^ = 13.65, *p* = 0.001, OR = 6.59; 95% CI 2.5–20.18). (4) Conclusion: Half of the patients investigated by using cardiac CT had a calcium score of zero. Predictors for coronary calcium scores in relation to risk factors varied. For the very severe coronary calcification level, the strongest predictor was the presence of smoking and diabetes, which increased the odds for very severe calcification by 13.46 times. Patients who had multiple cardiovascular risk factors, hypertension, diabetes, and smoking were 9.18 times more likely to have very severe calcification.

## 1. Introduction

Cardiovascular disease (CVD) is the leading cause of global mortality and morbidity in both developed and developing countries. Coronary artery disease (CAD) is a heart condition in which plaque builds up on the walls of the coronary arteries through a process called atherosclerosis. This leads to the narrowing of the coronary lumen with reduced blood flow to the myocardium and possible myocardial ischemia, major cardiac events, and even death. CAD and stroke account for more than 75% of CVD deaths [1,2,3].

The coronary artery calcium (CAC) score determined via computed tomography (CT) is a robust and well-established method for the early detection of CAD [1,4], and it is a sensitive marker of calcified atherosclerosis that correlates well with atherosclerotic plaque burden [1,5].

In the European ESC guidelines for the prevention of cardiovascular diseases, Romania is ranked as a very high-risk country [6], as well as in the European statistics on mortality from diseases of the circulatory system, where circulatory diseases are responsible for 50–60% of deaths [7]. However, data regarding the coronary CT calcium score and cardiovascular risk factors are limited; to our knowledge, this study is the first to report the association between coronary calcification levels assessed via cardiac CT and conventional cardiovascular risk factors in the southeastern region of the country. However, there was a study from the northwestern region of the country that assessed the association of cardiovascular risk factors and CAD using the Coronary Artery Disease Reporting Classification in 475 patients [8]; there were also other studies on the evaluation of patients with diabetes using cardiac CT [9,10], as well as the coronary CT calcification in patients with coronary artery anomalies [11].

Prevention and early detection of CAD are of critical importance; CAC is a subclinical marker and a powerful tool in the assessment of CAD, recommended primarily for risk stratification in asymptomatic patients [1,2,12] but also for risk assessment in symptomatic patients [13]. Current CAC guidelines recommend the use of non-contrast CT for the evaluation of CAC in asymptomatic patients, as it allows direct visualization of coronary atherosclerosis and improves the clinical risk assessment [1,2,14]. At the same time, coronary computed tomography angiography (CCTA) is increasingly used to evaluate symptomatic and high-risk patients for CAD [2,15,16].

Calcified plaque burden (CB) has an established value in predicting major cardiovascular events [15]; high CAC scores have been associated with an increased burden of atherosclerosis and stenosis and have been used for coronary heart disease (CHD) risk stratification and predicting the negative adverse effects of CHD [2,5,17,18]. CAC is now established as a reliable tool in the assessment of myocardial infarction risk, coronary mortality, and all-cause mortality stratification [1,2,4]. In addition, CCTA can also assess the anatomical location of plaques and the functional significance of CAD and provides information that can be used to guide preventive therapy [2,19].

CAD is a multifactorial disease; therefore, it is more clinically effective to evaluate it simultaneously, considering several risk factors associated with CAD [1]. To date, several studies have established the association of CAC with conventional cardiovascular disease risk factors and have also evaluated risk prediction models, biological markers, and imaging means of CVD screening [1,13,15,20,21]. Patients with more risk factors, for example, three factors compared to zero, one, or two, also had higher CAC scores; moreover, CAC has been reported to be an independent predictor of CAD risk [1,13,15,22].

The aim of this study was to establish the coronary calcium score in a group of patients investigated via cardiac CT and to determine the association between the CAC score and the presence of cardiovascular disease risk factors.

## 2. Materials and Methods

### 2.1. Study Design and Population

A retrospective pilot study was conducted, which included patients who underwent cardiac CT between February 2021 and April 2023 in the Radiology–Medical Imaging Clinical Center of the Carol Davila Military Central Emergency University Hospital, Bucharest, Romania.

The inclusion criteria were patients over 18 years of age who were recommended by their attending physician to undergo coronary computed tomography angiography (CCTA), patients known to have CAD to evaluate the degree of coronary involvement for better patient management, patients with a high risk of CAD, symptomatic patients with atypical angina, and patients with typical angina and other equivocal noninvasive tests.

Exclusion criteria were patients with acute coronary symptoms showing elevated biological cardiac markers or specific EKG changes of ischemia, patients with radiological contraindications, mainly pregnancy, and patients with contraindications to the administration of contrast agents. Of the 264 patients who underwent coronary CT angiography according to the recommendation of the attending physician, 222 were included, and the rest were excluded due to insufficient data in the patients’ medical records.

This study was conducted in accordance with the Declaration of Helsinki and approved by the Ethics Committee of Dr. Carol Davila’s Central Military Emergency University Hospital, Bucharest, Romania, No. 433/12.01.2021.

### 2.2. Methodology

Patients were clinically evaluated by the attending physician. Laboratory tests for serum creatinine were evaluated before CT examination. All patients underwent cardiac CT calcium score examination.

Data regarding patient clinical examination and laboratory analyses were retrieved from patients’ medical records; the presence of CVD risk factors and cardiac symptoms was established.

The Agatston calcium score was used to establish the degree of coronary artery calcification.

### 2.3. Medical Investigations

#### 2.3.1. Cardiac Computed Tomography

A CT system Revolution Evo 128-slice (GE Healthcare, Milwaukee, WI, USA) was used for scanning. The cardiac acquisition protocol used ECG monitoring, retrospectively, with continuous data acquisition during the cardiac cycle.

Patient preparation included routine premedication with an oral betablocker under the supervision of the attending physician to decrease the heart rate below 65 beats/minute, with intravenous supplementation during the examination when necessary. At the beginning of the examination, as indicated by the attending cardiologist, sublingual nitroglycerin was administered.

The cardiac CT investigation procedure included a first non-contrast CT scan for the evaluation of coronary calcium scores. Contrast scanning for coronary lumen analysis involved the administration of 300–400 mg/mL iodine-based contrast at high flow rates of 5.5 mL/s, a total of 60 mL iodinated contrast, followed by a 30 mL mixture of contrast agent with serum.

Image post-processing and analysis were performed using a GE Medical System AW Volume Share 7 with SmartScore version 4.0 (GE Medical Systems, Buc, France). The evaluation was performed using source images in the axial plane and planar and curved reconstructions of the coronary arteries. The Agatston calcium score was calculated semi-automatically in all patients.

#### 2.3.2. Agatston Calcium Score

The Agatston calcium score is a specific method for evaluating calcium deposits in the coronary arteries (CAC) that occur in the atherosclerotic process of plaque formation. It is a rapid tomography scan that is performed at the beginning of the CCTA examination before the administration of the contrast substance. Based on the attenuation density in Hounsfield units measured semi-automatically, a total coronary artery calcium score is obtained. The score is expressed by absolute numerical values that are divided into groups, with each group corresponding to a level of cardiovascular risk. Calcium score values are 0 UA no calcification, 1–10 UA minimal calcification, 11–100 UA mild calcification, 101–400 UA moderate calcification, 401–1000 UA severe calcification, and over 1000 UA extensive very severe calcification [23,24].

### 2.4. Risk Factors’ Definitions

Blood pressure (BP) was classified according to ESC/ESH Guidelines for the management of arterial hypertension 2018 [25]: normal BP includes systolic values < 130 mmHg and diastolic BP < 85 mmHg, and high normal BP includes values between 130 and 139 mmHg systolic and 85 and 89 mmHg diastolic BP. Grade I hypertension includes values between 140 and 159 mmHg systolic and/or 90 and 99 mmHg diastolic BP; grade II hypertension includes values between 160 and 179 mmHg systolic and/or 100 and 109 mmHg diastolic BP; grade III hypertension includes values ≥ 180 mmHg systolic and/or ≥110 mmHg diastolic BP. Hypertension was defined as resting systolic BP ≥ 140 mmHg systolic and/or ≥90 mmHg diastolic BP, a history of hypertension, or taking BP-lowering medication.

Patient obesity was established using the body mass index (BMI) formula, BMI = kg/m^2^, with BMI of 30 or higher being in the obesity range.

Dyslipidemia was defined as one or more high lipid profile parameters: low-density lipoprotein cholesterol (LDL-C) ≥ 160 mg/dL, total cholesterol (TC) ≥ 200 mg/dL, high-density lipoprotein cholesterol (HDL-C) < 40 mg/dL, triglycerides ≥ 200 mg/dL, taking lipid-lowering medication, or a history of dyslipidemia.

Diabetes mellitus was established as a history of diabetes, taking hypoglycemic medication, or a value of fasting blood glucose ≥ 120 mg/dL.

Smoking patients were current smokers or those with a history of smoking.

### 2.5. Statistical Analysis

The data were analyzed in IBM SPSS Statistics version 20 (IBM, Chicago, IL, USA). For the qualitative data, the frequency and the percentage were used, and in order to identify the risk factors, several chi-square tests and odds ratios were employed. All the statistical tests were considered statistically significant at a *p* value < 0.05. In order to have a more powerful approach, the odds ratio (OR) was used along with the confidence intervals.

## 3. Results

### 3.1. Evaluation of Patients via Cardiac CT and Establishing the Presence of Coronary Atherosclerotic Disease Using the Agatston Calcium Score

This study included 222 subjects, 115 (51.8%) females, with ages between 21 and 84 years and a mean age of 58.16 (+/− 12.56) years. Of these, 174 (78.4%) were patients over 50 years old.

Among the patients included in this study, 66.7% had hypertension, 63.5% had dyslipidemia, 30.2% were obese, 25.2% were smokers, and 19.5% had diabetes mellitus.

All subjects underwent cardiac CT for calcium score evaluation, the Agatston calcium score was assessed, and coronary artery calcification was present in 111 (50%) patients.

**Figure 1:**
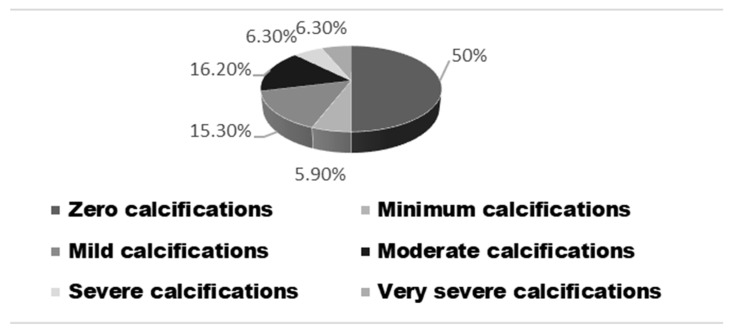
The distribution of the Agatston calcium scores.

According to the Agatston calcium score, 111 (50%) patients had a zero calcium score, 13 (5.9%) had a minimum calcification with calcium score values between 1 and 10 UA, 34 (15.3%) had mild calcium score values between 11 and 100 UA, 36 (16.2%) had moderate calcium score values between 101 and 400 UA, 14 (6.3%) had severe calcium score values between 401 and 1000 UA, and 14 (6.3%) had severe calcium score with values above 1000 UA (Figure 1).

### 3.2. Characteristics of Patients with Coronary Atherosclerotic Disease

In the group of patients with a zero calcium score, 72 (64.9%) were patients over 50 years old, and 63 (56.8%) were female. According to Table 1, in terms of percentages, there are some differences between genders and their symptoms depending on their calcium scores. For instance, in the case of the absence of calcium (Category 0), male patients registered 24.3% of symptoms, while female patients registered 42.3% of symptoms. In addition, female patients registered a higher percentage of symptoms in what are concerned the calcium categories 1–10 (61.5%), 11–100 (47.1%), and 101–400 (47.2%), while the male patients registered more symptoms in the category > 1000 of the calcium score (64.3%).

There was a statistically significant difference between the ages of patients under 50 and over 50 and the different levels of calcification (chi-square = 24.39, *p* = 0.001). Thus, there were differences between patients aged under 50 and over 50 years at the level without calcification (39 vs. 72) and moderate calcification (2 vs. 34).

There was no statistically significant difference between the sex of the patients and the different levels of calcification (chi-square = 7.52, *p* = 0.18).

### 3.3. Determining the Association between Cardiovascular Risk Factors and Coronary Atherosclerotic Disease

#### 3.3.1. Establishing the Presence of Arterial Hypertension and the Association with the Agatston Calcium Score

**Figure 2:**
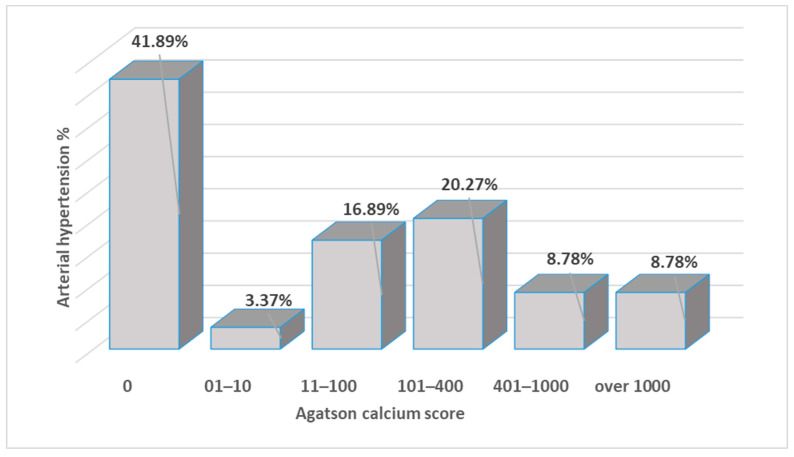
The distribution of the hypertensive patients and the calcification scores.

In the sample of patients, there were 148 (66.7%) patients with elevated blood pressure. There were statistically significant associations between hypertensive patients and calcification levels (chi-square = 24.35, *p* = 0.001). Thus, there were hypertensive patients without calcification (62 vs. 49), with moderate calcification (30 vs. 6), severe calcification (13 vs. 1), and very severe calcification (13 vs. 1), as depicted in Figure 2.

#### 3.3.2. Establishing the Presence of Obesity and the Association between Obesity and Coronary Calcium Score

In the sample of patients, 67 (30.2%) were obese with a BMI over 30. There was no statistically significant difference between the fact that the patients were obese and the different levels of calcification (chi-square = 0.99, *p* = 0.96).

#### 3.3.3. Establishing the Presence of Dyslipidemia and the Association with Coronary Calcium Score

Of the patient sample, 81 (36.5%) patients did not have dyslipidemia, and 141 (63.5%) patients had dyslipidemia.

There was a statistically significant difference between patients with dyslipidemia and the different levels of calcification (chi-square = 15.80, *p* = 0.007). Thus, patients with moderate calcification had dyslipidemia (30 vs. 6).

#### 3.3.4. Determining the Presence of Diabetes and the Association with the Coronary Calcium Score

In the group of patients, 43 (19.4%) had diabetes. There was a statistically significant difference between patients with diabetes and the different levels of calcification (chi-square = 25.20, *p* = 0.001). Thus, patients with diabetes did not present calcification (without calcification—10 vs. 101).

#### 3.3.5. Establishing the Presence of Smoking and the Association with Coronary Atherosclerotic Disease

Of the patient sample, 56 (25.2%) patients were smokers. There was a statistically significant difference between the patients who were smokers and the different levels of calcification (Chi-square = 12.16, *p* = 0.03). Thus, smokers had mild calcification (4 vs. 30).

#### 3.3.6. Establishing the Associations between Coronary Artery Calcification Levels and the Presence of Cardiovascular Risk Factors

There was a statistically significant association between the mild level of calcification and the age of patients over 50 years (X^2^ = 3.88, *p* = 0.04). As such, age over 50 years was considered a risk factor for mild calcification (OR = 3.25; 95% CI 0.94–11.14). Patients over the age of 50 were 3.25 times more likely to present mild calcification.

There was a statistically significant association between the moderate level of calcification and the age of patients over 50 years (X^2^ = 6.54, *p* = 0.01). An age over 50 years was considered a risk factor for moderate calcification (OR = 5.58; 95% CI 1.29–24.16). Patients over the age of 50 were 5.58 times more likely to have moderate calcification.

There was a statistically significant association between the moderate level of calcification and the presence of dyslipidemia (X^2^ = 7.28, *p* = 0.007). As such, dyslipidemia was considered a risk factor for moderate calcification (OR = 3.37; 95% CI 1.34–8.51). Patients with dyslipidemia were 3.37 times more likely to present moderate calcification.

There was a statistically significant association between the moderate level of calcification and the presence of hypertension (X^2^ = 5.37, *p* = 0.02). Hypertension was considered a risk factor for moderate calcification (OR = 2.88; 95% CI 1.14–7.27). Patients with hypertension were 2.88 times more likely to have moderate calcification.

There was a statistically significant association between the severe level of calcification and the presence of hypertension (X^2^ = 4.61, *p* = 0.03). Hypertension was considered a risk factor for severe calcification (OR = 7.03; 95% CI 0.90–54.81). Patients with hypertension were 7.03 times more likely to present severe calcification.

There was a statistically significant association between the very severe level of calcification and the presence of diabetes (X^2^ = 13.65, *p* = 0.001). Diabetes was considered a risk factor for very severe calcification (OR = 6.59; 95% CI 2.5–20.18). As such, patients with diabetes were 6.59 times more likely to have very severe calcification.

There was a statistically significant association between the very severe level of calcification and the presence of hypertension (X^2^ = 4.61, *p* = 0.03). Hypertension was considered a risk factor for very severe calcification (OR = 7.03; 95% CI 0.90–54.81). Patients with hypertension were 7.03 times more likely to develop very severe calcification.

There was a statistically significant association between the very severe level of calcification and smoking (X^2^ = 8.07, *p* = 0.004). Smoking was considered a risk factor for very severe calcification (OR = 4.44; 95% CI 1.47–13.44). Smokers were 4.44 times more likely to develop very severe calcification.

Taking into account the risk factors (age over 50 years, male sex, hypertension, dyslipidemia, obesity, smoking, diabetes mellitus), specifically the number of risk factors, among patients with a calcium score > 1000, 42.9% had five risk factors, 28.6% had four risk factors, 21% had six risk factors at the same time, 7.1% had seven risk factors, and no patient presented less than four risk factors.

Among the patients with a calcium score of 401–1000, 50% of the patients had four concomitant risk factors, 28.6% had five risk factors, 14.3% had three risk factors, and 7.1% had two risk factors, and no patient presented more than five risk factors or less than two.

Among the patients with a calcium score of 101–400, 33.3% of the patients had four concomitant risk factors, 27.8% had three risk factors, 13.9% had five risk factors, and no patient presented zero or one risk factors.

Among the patients with a calcium score of 11–100, 38.2% presented simultaneously three risk factors, 29.4% of patients presented four risk factors, 14.7% presented five risk factors, and no patient presented zero, one, or seven risk factors.

Among the patients with a calcium score of 1–10, 30.8% presented three concomitant risk factors, 23.1% of the patients presented two risk factors, 23.1% presented five risk factors, and no patient presented zero or seven risk factors.

Among the patients with a calcium score of 0, 28.8% had three risk factors, 26.1% had two risk factors, and 16.1% had four risk factors.

Considering the number of risk factors, all patients with a calcium score above 1000 had more than three risk factors. Among the patients with a calcium score of 401–1000, 78.6% had more than three risk factors, 63.9% of the patients with a calcium score of 101–400 had more than three risk factors, 44.1% of the patients with a score of calcium 11–100 had more than three risk factors. Among the patients with zero calcium score, 72.1% had less than three risk factors.

#### 3.3.7. The Association between Coronary Artery Calcification Levels and the Presence of Several Cardiovascular Risk Factors

- There was a statistically significant association between the moderate level of calcification and the presence of dyslipidemia and hypertension (X^2^ = 9.93, *p* = 0.002). As such, the presence of both dyslipidemia and arterial hypertension was considered a risk factor for moderate calcification (OR = 3.37; 95% CI 1.53–7.38). Therefore, patients with dyslipidemia and arterial hypertension were 3.37 times more likely to have moderate calcification;
- There was a statistically significant association between the moderate level of calcification and the presence of dyslipidemia and age over 50 years (X^2^ = 9.38, *p* = 0.002). As such, the presence of both dyslipidemia and age over 50 years was considered a risk factor for moderate calcification (OR = 3.50; 95% CI 1.51–8.08). Patients with dyslipidemia and an age over 50 years were 3.50 times more likely to present moderate calcification;
- There was a statistically significant association between the moderate level of calcification and the presence of hypertension and age over 50 years (X^2^ = 11.00, *p* = 0.001). The presence of both hypertension and an age over 50 years was considered a risk factor for moderate calcification (OR = 4.05; 95% CI 1.69–9.71). Patients with hypertension and over 50 years old were 4.05 times more likely to present moderate calcification;
- There was a statistically significant association between the moderate level of calcification and the presence of dyslipidemia, hypertension, and the age of patients over 50 years (X^2^ = 13.40, *p* = 0.001). As such, the presence of all three factors, dyslipidemia, hypertension, and being over 50 years of age, was considered a risk factor for moderate calcification (OR = 3.94; 95% CI 1.82–8.51). Therefore, patients with dyslipidemia, arterial hypertension, and over 50 years old were 3.94 times more likely to have moderate calcification;
- Regarding the presence of very severe calcification, there was a statistically significant association between the presence of smoking and diabetes (X^2^ = 20.12, *p* = 0.001). The presence of both smoking and diabetes was considered a risk factor for very severe calcification (OR = 13.46; 95% CI 3.27–55.46). Smokers with diabetes were 13.46 times more likely to present very severe calcification;
- There was a statistically significant association between the very severe level of calcification and the presence of smoking and hypertension (X^2^ = 11.38, *p* = 0.001). The presence of both smoking and hypertension was considered a risk factor for very severe calcification (OR = 5.71 95% CI 1.8–17.41). Smokers with hypertension were 5.17 times more likely to present very severe calcification;
- There was a statistically significant association between the very severe level of calcification and the presence of diabetes and hypertension (X^2^ = 10.85, *p* = 0.001). The presence of both diabetes and hypertension was considered a risk factor for very severe calcification (OR = 5.50 95% CI 1.80–16.74). As such, patients with diabetes and hypertension were 5.50 times more likely to present very severe calcification;
- There was a statistically significant association between the very severe level of calcification and the presence of smoking, diabetes, and hypertension (X^2^ = 11.59, *p* = 0.001). As such, the presence of all three factors, smoking, diabetes, and hypertension, was considered a risk factor for very severe calcification (OR = 9.18; 95% CI 2.02–41.68). Therefore, smokers with diabetes and hypertension were 9.18 times more likely to have very severe calcification.

### 3.4. Determining the Presence of Cardiac Symptoms and the Association with the Coronary Atherosclerosis

From the patient sample, 164 (73.9%) had cardiac symptoms. There was no statistically significant difference between the patients with cardiac symptoms and the levels of calcification (chi-square = 7.73, *p* = 0.17).

## 4. Discussion

The CAC score is a marker directly related to known atherosclerosis risk factors that correlate well with atherosclerotic plaque burden and can be accurately assessed via computed tomography using the Agatston calcium score compared to other cardiac risk factors-based paradigms [1,5,13,15].

In our study, according to the Agatston calcium score, coronary artery calcification was present in 111 (50%) of the patients, similar to other studies in the scientific literature, where the CCTA revealed that approximately 40–55.4% of the studied patients had a positive CAC score [12,13], but there have been studies reporting a higher prevalence of CAC score of up to 70% [15].

A significant finding was that half of the patients investigated via cardiac CT had a calcium score of zero. According to Blaha et al., participants with CAC = 0 had the lowest proportion of CHD events, other negative tests, and clinical characteristics such as age, sex, smoking, diabetes, total and HDL cholesterol, and blood pressure did not review the post-test risk as much as CAC [20]. Additional information is needed to define prevention strategies. To have the best risk prediction tools, the use of CAC from non-contrast cardiac computed tomography is superior to conventional risk factors and serum biomarkers in primary prevention [26].

High CAC scores above 400 UA were present in 12.6% of patients, similar to other studies that reported rates of 15.9% and generally from 10% to 20% [2,19,27].

The findings of this study revealed that the mild level of calcification was statistically significantly associated with patients over 50 years of age (X^2^ = 3.88, *p* = 0.04). Being over 50 years of age increased the odds of having a mild level of calcification by 3.25 times (OR = 3.25; 95% CI 0.94–11.14).

The moderate level of calcification was statistically significantly associated with being over 50 years of age, having dyslipidemia, and having hypertension. Being over 50 years of age was the strongest predictor for the moderate level of calcification. Being over 50 years of age increased the odds of having a moderate level of calcification by approximately 5.58 times (OR = 5.58; 95% CI 1.29–24.16). The second predictor for a moderate level of calcification was dyslipidemia. Dyslipidemia increased the odds of having a moderate level of calcification by approximately 3.37 times (OR = 3.37; 95% CI 1.34–8.51). The third strongest predictor for a moderate level of calcification was hypertension. Having hypertension increased the odds of having a moderate level of calcification by approximately 2.88 times (OR = 2.88; 95% CI 1.14–7.27).

The severe level of calcification was statistically significantly associated with the presence of hypertension. The presence of hypertension increased the odds of having a severe level of calcification by approximately 7.03 times (OR = 7.03; 95% CI 0.90–54.81).

In this study, the very severe level of calcification was statistically significantly associated with the presence of smoking, diabetes, and hypertension. Hypertension was the strongest predictor for the very severe level of calcification. Having hypertension increased the odds of having a very severe level of calcification by approximately 7.03 times (OR = 7.03; 95% CI 0.90–54.81). The second predictor for a very severe level of calcification was diabetes. Diabetes increased the odds of having a very severe level of calcification by approximately 6.59 times (OR = 6.59; 95% CI 2.50–20.18). The third strongest predictor for very severe levels of calcification was smoking. Being a smoker increased the odds of having a very severe level of calcification by approximately 4.44 times (OR = 4.44; 95% CI 1.47–13.44).

Overall, the findings are consistent with findings in the scientific literature. For instance, Wada et al. also found that patients diagnosed with coronary stenosis via CCTA were older, current or past smokers who had hypertension or diabetes; the difference was the association with the male sex and low levels of HDL and total cholesterol in the context of higher rates of statin use [22]. Kaolawanich et al. found that high CAC >400 was significantly associated with hypertension [2]. Similar to the findings in the present study, in other studies, patients with high blood pressure were reported to have elevated CAC scores [28]. High CAC scores were also associated with being over 65 years of age and taking antithrombotic or statin medication. Additional predictors were diabetes, hyperlipidemia, and a history of vascular diseases. Age was one of the strongest predictors for the CAC score, with asymptomatic patients over 65 years of age having six times higher CAC scores than younger patients under 45 years of age [2]. Many other studies, including Gordon et al., found age to be associated with calcified atherosclerotic burden; additional risk factors in this study were sex and statin use [15]. The Multi-Ethnic Study of Atherosclerosis (MESA) [29] found that age and all conventional risk factors for CHD, such as diabetes, smoking, blood pressure, and cholesterol, were associated with CAC, confirming the findings of our study, and in addition to the findings of this study, sex and race–ethnicity were significantly associated with CAC. Min et al. found that being over 40 years of age, male sex, higher rates of hypertension, dyslipidemia, and diabetes were associated with positive CAC values, and certain traditional cardiovascular risk factors such as age over 40 years, diabetes, and smoking acted as independent factors in the conversion of zero CAC to positive values. Moreover, the level of CAC at the time of CAD diagnosis was the critical factor among other risk factors in determining the progression of atherosclerosis [5]. Yazdi et al. found the most prevalent conventional risk factors to be lipid disorder (30.2%), diabetes mellitus (17.9%), and hypertension (9%), which were more likely to be associated with positive CAC score [13], which is similar to our results, except for hypertension which had a higher prevalence. However, the association between the presence of cardiovascular risk factors and the levels of coronary artery calcification varied as follows [13]: mild CAD was associated with diabetes and hypertension, unlike in our study, where it was associated with age over 50 years. For moderate CAC, diabetes was the strongest predictor, smoking was the second predictor, and sex was the third predictor, in contrast to the present study where there was a statistically significant association between the moderate level of calcification and the presence of dyslipidemia, hypertension, and being over 50 years of age. CAC > 400 was associated with smoking, diabetes, and family history, as in our study, where very severe CAC was significantly associated with smoking and diabetes. Similar to our study, BMI was not a significant predictor for CAC [13]. Other studies that evaluated the association between the presence of cardiovascular risk factors and the levels of coronary artery calcification found a significant association between the mild level of CAC and the presence of hypertension and the moderate level of CAC and smoking, which was different from our findings. Finally, similarities were present in severe levels of CAC that were associated with diabetes, smoking, and hypertension, and additionally with obesity [12].

Regarding the analysis of the sum of cardiovascular risk factors, all patients with a calcium score above 1000 had more than three risk factors (*p*< 0.001). Half of the patients with severe calcifications had simultaneously four cardiovascular risk factors, and 78.6% had more than three cardiovascular risk factors. On the other hand, among the patients with zero calcium score, 72.1% had less than three risk factors. Thus, it may be concluded that the higher the number of risk factors, the higher the calcification score.

In addition to studies in the scientific literature, the present study established the association between several cardiovascular risk factors and coronary artery calcification levels. As such, the presence of hypertension and age over 50 years had the strongest association with a moderate level of calcification. Patients with hypertension and age over 50 years were 4.05 times more likely to present moderate calcification (OR = 4.05; 95% CI 1.69–9.71). The moderate level of calcification was also statistically associated with the presence of dyslipidemia and age over 50 years. Patients with dyslipidemia and an age over 50 years were 3.50 times more likely to present moderate calcification (OR = 3.50; 95% CI 1.51–8.08). The moderate level of calcification was also statistically significantly associated with the presence of dyslipidemia and hypertension. Thus, patients with dyslipidemia and hypertension were 3.37 times more likely to present moderate calcification (OR = 3.37; 95% CI 1.53–7.38). Finally, the presence of all three factors, dyslipidemia, hypertension, and being over 50 years of age, was statistically associated with a moderate level of calcification. Patients with dyslipidemia, hypertension, and over 50 years of age were 3.94 times more likely to present moderate calcification.

This study brings additional information to the specialized literature regarding the presence of very severe levels of coronary calcification and associations with multiple cardiovascular risk factors. Regarding the presence of very severe calcification, the strongest statistically significant association was with the presence of smoking and diabetes. Smokers with diabetes were 13.46 times more likely to present very severe calcification (OR = 13.46; 95% CI 3.27–55.46). Secondly, the very severe level of calcification was statistically significantly associated with the presence of smoking and hypertension. Smoking patients with hypertension were 5.71 times more likely to present very severe calcification (OR = 5.71 95% CI 1.8–17.41). The very severe level of calcification was also statistically associated with the presence of diabetes and hypertension. Patients with diabetes and hypertension were 5.50 times more likely to present very severe calcification (OR = 5.50 95% CI 1.80–16.74). Data on the association of diabetes and smoking with increased levels of coronary artery calcification using CT introduce additional information, and Popa et al. found no association between diabetes and CAD [8]. Finally, the presence of all three factors, smoking, diabetes, and hypertension, was statistically significantly associated with the presence of very severe levels of calcification. Patients who presented all the risk factors, smoking, diabetes, and hypertension, were 9.18 times more likely to present very severe calcification (OR = 9.18; 95% CI 2.02–41.68).

CAC provides a personalized CAD risk assessment that can identify patients predicted to benefit most from treatment. Negative CVD risk markers help identify patients less suitable for preventive pharmacologic therapy. Evidence suggests that the CAC may also promote long-term adherence to aspirin and statin therapy, as well as exercise and diet, with a threefold higher likelihood of statin and aspirin use in patients evaluated for CAC [26,30].

Disadvantages of the CAC include radiation exposure, but for a CAC scan, the radiation exposure is approximately 1 mSv, equivalent to 120 additional days of ambient radiation exposure, and the potential benefits of preventing CHD mortality when CAC = 0 must be considered over a small increase in lifetime cancer risk. There is also the risk of incidental findings, the frequency of which is noted at 43 out of 1000 people tested for CAC, with only 1.2% of findings having clinical significance. The increased cost of follow-up is another concern, but resources are often directed to those with the highest risk of CVD, and people with CAC = 0 have lower downstream medical expenses than those without CAC testing [26].

Limitations of this study from an imaging perspective are the difficulty in obtaining quality images in patients who cannot perform respiratory commands or have heart rhythm irregularities during the examination.

The results of this study cannot be generalized for the entire population of Romania, primarily because it is a pilot study; so, the number of patients was relatively small, and the results were obtained for patients from a single medical center. Furthermore, the results cannot be generalized, as the included patients who underwent coronary CT were symptomatic or had CVD risk factors; so, the patient population distribution does not represent the CAC score with respect to asymptomatic patients at low CVD risk. Also, analyses of CAC in association with CAD-RADS were not performed.

Future research directions include continuing the study of a larger number of patients, increasing the number of CCTA investigations, and bringing more evidence to the data in the country.

## 5. Conclusions

A significant finding is that half of the patients investigated via cardiac CT in a high-cardiovascular-risk country had a calcium score of zero. Patients with positive CAC score values were more likely to be older and to have more risk factors for CAD, including hypertension, dyslipidemia, diabetes, and smoking, but predictors for CAC scores in relation to risk factors varied. Older age increased the odds of mild CAC, while hypertension, dyslipidemia, and older age increased the odds of moderate CAC, and severe CAC was associated with hypertension. This study established additional associations for very severe CAC; smokers with diabetes were 13.46 times more likely to have very severe calcification, and patients with all three risk factors, hypertension, diabetes, and smoking, were 9.18 times more likely to have very severe calcification.

## Figures and Tables

**Table 1:** The prevalence of the calcium score in the study subjects depending on sex and symptoms.

Calcium Score	Sex			
	Male (%)		Female (%)	
	Symptoms +	Symptoms −	Symptoms +	Symptoms −
0	24.3%	18.9%	42.3%	14.4%
1–10	23.1%	15.4%	61.5%	0.0%
11–100	29.4%	17.6%	47.1%	5.9%
101–400	38.9%	13.9%	47.2%	0.0%
401–1000	35.7%	21.4%	35.7%	7.1%
>1000	64.3%	14.3%	21.4%	0.0%

## Data Availability

The data presented in this study are available on reasonable request from the corresponding author (A.S.C.). The data are not publicly available due to ethical restrictions.
